# Positive Psychology Practices in Muslim Communities: A Systematic Review

**DOI:** 10.1007/s10943-025-02357-9

**Published:** 2025-06-14

**Authors:** Gazanfer Anlı

**Affiliations:** Department of Psychology, Faculty of Humanities and Social Sciences, Bursa Technical University, 16330 Yıldırım, Bursa, Turkey

**Keywords:** Positive psychology, Positive psychotherapy, Muslim community, Review

## Abstract

Psychology’s positive subfield emphasizes human well-being by concentrating on the strengths, virtues, and elements that promote it, as opposed to focusing solely on mental illness and disadvantage. Positive psychology interventions have gained international recognition, but their application and impact in non-Western societies, especially within Muslim communities, have not been thoroughly examined. Integrating positive psychology into psychotherapy practices within the Muslim community involves a distinctive blend of cultural, religious, and psychological factors. Practicing positive coping mechanisms through prayer and social support from the community is associated with better mental well-being. This study was carried out with the aim of providing a synthesis of articles on the practice of positive psychology in Muslim communities. Based on the search strategy as well as inclusion and exclusion criteria, four studies were chosen for further examination. These studies encompassed a wide range of populations, such as nurses and university students in Indonesia, humanitarian workers in the Philippines, and students in Malaysia. Participants across these four studies reported improvements in their psychological well-being, including heightened happiness, decreased stress levels, and increased post-traumatic growth as a result of positive psychology and Islamic-influenced approaches. This study provides a comprehensive analysis of the impact and cultural appropriateness of positive psychology interventions among Muslim populations. It seeks to pinpoint optimal methodologies and potential avenues for further investigation by reviewing multiple existing studies.

## Introduction

The positive branch of psychology, which concentrates on the abilities and admirable qualities that allow individuals and communities to flourish, has gained popularity in various therapeutic environments (Montiel et al., 2021). In contrast, psychotherapy offers scientifically supported treatments for managing psychological distress and facilitating recovery. Incorporating principles from positive psychology into psychotherapy can boost the treatment’s effectiveness and promote a more comprehensive approach to mental health and well-being.

As Tan (2023) describes, the PERMA model, which consists of Positive Emotion, Engagement, Relationships, Meaning, and Accomplishment, provides a fundamental structure for numerous positive psychological interventions (PPIs). This framework is particularly relevant in Muslim communities, where community and relational aspects are deeply intertwined with individual identity and overall well-being, as noted by Basurrah et al. (2021).

In this context, positive psychology can yield significant benefits for the Muslim community, particularly as mental health issues are often intertwined with cultural and religious values (Tanhan & Francisco, 2019). Therefore, the integration of spiritual and religious components into therapeutic practices is essential to effectively address the mental health needs of Muslim individuals (Khait & Lazenby, 2021; Saleem et al., 2021).

### Positive Psychology Interventions in Muslim and Non-Muslim Communities

Research has provided evidence that positive psychology interventions are effective in multiple settings. A study of the “Happy Family Kitchen” program in Hong Kong found that family-focused public–private initiatives could enhance subjective happiness and health-related quality of life (Ho et al., 2016a). Culturally sensitive interventions that focus on forgiveness and empathy have yielded positive results in improving well-being among Muslim teenagers, according to research by Yaghoobi and Moghadam (2019). Positive psychology online interventions are also being used. The COVID-19 pandemic has expedited the uptake of online interventions, demonstrating potential benefits for psychological well-being across various populations, such as Muslim communities (DuPont et al., 2022). Online platforms offer accessibility and flexibility, enabling people to interact with patient-public involvement initiatives in a way that considers cultural differences and individual circumstances (Mitchell et al., 2010). Research has shown that online PPIs can effectively boost positive emotions and decrease the symptoms of anxiety and depression (Feizi, 2024).

A comprehensive literature review showed a rising interest in using PPI among Arab and Muslim populations. A systematic review carried out by Basurrah et al. demonstrated the efficacy of PPIs in Arab countries, showing considerable enhancements in well-being and mental health outcomes (Basurrah et al., 2022). Interventions that focus on the strengths and involvement of the community produce positive outcomes, which are consistent with the cultural values of these communities. Community-level initiatives have shown a significant impact in promoting resilience and social unity among Muslim communities. According to Montiel et al., community-based participatory initiatives can notably improve overall quality of life by fostering functioning and decreasing symptom-related issues (Montiel et al., 2021). Studies have found that programs that include community rituals, social support systems, and collective expressions of gratitude can increase communal bonds and boost overall mental well-being (Ho et al., 2016b). Mindfulness practices, now widely used in positive psychology, can be modified to complement Islamic practices like meditation and introspection (Tanhan, 2019). Studies have found that mindfulness can lead to better mental health results through the encouragement of emotional control and a decrease in anxiety levels (Arfken & Ahmed, 2016; Omu et al., 2012). Therapists can provide culturally sensitive interventions that improve well-being by incorporating mindfulness techniques that align with Islamic principles (Altalib et al., 2019; Mir et al., 2019).

The key distinctions between positive psychology interventions created for Muslims and those designed for non-Muslims lie in cultural context, religious beliefs, and the incorporation of spiritual elements into therapeutic approaches. The emphasis on strengths, virtues, and well-being in positive psychology can differ widely between Muslim and non-Muslim groups due to varying values, worldviews, and coping strategies shaped by their distinct faiths, which significantly impact the application of these principles.

Initially, Muslim positive psychology interventions systematically incorporate Islamic teachings and core principles into their underlying frameworks. Therapists typically take spiritual beliefs into account when creating treatment plans. Interventions may comprise practices grounded in Islamic spirituality, like prayer, trust in God (tawakkul), and the notion of gratitude (shukr), which serve as mechanisms to improve well-being (Thomas & Barbato, 2020). These interventions draw upon religious coping mechanisms that are highly relevant in Muslim communities (Uddin et al., 2022), building a therapeutic atmosphere that acknowledges and validates the client’s religious background. In contrast, non-Muslim positive psychology interventions usually do not have this spiritual element and are frequently secular in nature, concentrating on universal psychological concepts such as resilience and emotional intelligence without explicit connections to religious beliefs.

In addition, the framework of positive psychology among Muslims frequently highlights the importance of community and family ties, which are deeply rooted in Islamic tradition. Interventions in Muslim communities might utilize community-based support systems, including congregational prayer sessions (“salah”) or community service activities (such as “zakat” or almsgiving), as therapeutic methods to enhance mental health (Abdulkerim & Li, 2022). In contrast, many Western approaches in positive psychology often focus on individualistic solutions for achieving well-being. Understanding the larger social context in which individuals live is crucial for Muslims, meaning that psychotherapy methods that involve community participation can lead to a more significant therapeutic outcome.

Cognitive behavioral therapy (CBT) has been successfully modified for Muslim communities; however, the modifications demonstrate the incorporation of Islamic principles rather than a straightforward implementation of the original model (Haque et al., 2016). For example, Islamic adaptations of CBT may involve incorporating concepts related to faith and spiritual acceptance that are relevant to Muslim clients. Reframing negative thoughts through a faith-based perspective may involve interpreting personal struggles as opportunities for growth and resilience, as perceived from a spiritual viewpoint. Conversely, non-Muslim interventions might be limited by their adherence to secular methods that do not take into account spiritual or faith-based beliefs.

In sum, the key difference between positive psychology interventions for Muslims and those for non-Muslims hinges on the inclusion of cultural, spiritual, and community-based practices that hold significant meaning for the Muslim community. Combining Islamic beliefs with psychological concepts, positive psychology for Muslims acknowledges that an individual’s faith plays a vital role in their overall well-being. It highlights the importance of communal support, spiritually grounded coping methods, and culturally tailored approaches, which are frequently lacking in non-Muslim frameworks that often prioritize secularism or individualism. It is crucial for mental health specialists to deliver culturally sensitive care that acknowledges and complements the spiritual aspects of their patients’ lives in order to grasp these distinctions.

### Religion, Spirituality, and Mental Health in the Muslim Community

For many Muslims, religion and spirituality play a crucial role in shaping their lives. These aspects significantly influence how they approach challenges and manage their mental health, as highlighted by Casey et al. (2020). Research has shown that increased religiosity is linked to improved mental health and overall well-being among Muslims (Han et al., 2021; Mitha, 2020). Additionally, employing positive religious coping mechanisms, such as finding solace in prayer and support from one’s community, has been associated with enhanced mental health outcomes (Diaków & Goforth, 2021; Lowe et al., 2019). Incorporating these elements into psychotherapy can strengthen the therapeutic relationship and facilitate the healing process. Cultural background significantly influences the success of psychological treatments. In Muslim communities, values such as collectivism, spirituality, and community support are of the utmost importance (Hendriks et al., 2018). Studies suggest that culturally tailored interventions aligned with these values have a higher chance of being successful (Muller-Dugic, 2024). Islamic teachings place a strong emphasis on practices of gratitude, which have been shown to improve well-being and promote a sense of community (Yurayat & Seechaliao, 2021). This requires grasping the cultural and religious background of clients and modifying therapy techniques in response. Integrating Islamic teachings and values into therapy can make clients feel more understood and at ease (Alhomaizi et al., 2018; Bagasra & Mackinem, 2014; Tangsangwornthamma et al., 2018). Therefore, educating mental health professionals to acknowledge and respect the distinct cultural nuances within Muslim communities is essential for building trust and participation (Ali et al., 2022; Mullick et al., 2013).

### Integrating Islamic Principles into Positive Psychology and Psychotherapy Interventions

Including Islamic principles in psychotherapy can improve the significance and success of therapeutic treatments for Muslim patients (Martin, 2015). The healing process involves acknowledging the significance of faith, prayer, and community (Ali et al., 2023). Mental health discussions can be framed using Islamic teachings, enabling therapists to connect clients’ experiences to their faith (Nadir, 2024; Sabry & Vohra, 2013). Islamic teachings place great significance on the importance of one’s mental and spiritual health. Core Islamic values like “tawakkul” (trust in God), “sabr” (patience), and “shukr” (gratitude) are integral components of Islamic tradition, providing essential tools for overcoming obstacles and cultivating resilience (Hassan, 2021).

Several theoretical frameworks have influenced the connection between Islam and positive psychology, focusing on the convergence of spiritual and mental well-being. The concept of resilience is a key theme in the intersection of Islam and positive psychology, especially as it pertains to students. Aprilianti’s study demonstrates that incorporating Islamic psychological principles can boost resilience—a finding that aligns with established theoretical models but is frequently overlooked in Western psychology research (Aprilianti, 2024). Incorporating diverse spiritual perspectives is crucial for positive psychology to gain a more comprehensive understanding of well-being. The research highlights the significance of incorporating spiritual values to enhance academic resilience, revealing a critical connection between religious beliefs and psychological adaptability.

In this context, Saritoprak and Abu-Raiya also explore the Islamic framework of positive psychology in more detail. This study highlights key elements crucial to a fulfilling life as presented through Islamic principles, including appreciation, empathy, and significant connections, which are vital for attaining mental well-being according to Islamic teachings (Saritoprak & Abu-Raiya, 2023). The underlying principles of Islam play a significant role in promoting desirable mental states in people, which is in line with the overall aims of positive psychology to boost life satisfaction and mental well-being.

Research into psychological experiences has shown that interventions based on Islamic philosophy can make a notable contribution to well-being, especially in healthcare environments. A study carried out by Komariah et al. demonstrates the psychological advantages gained from the application of Islamic philosophy among women afflicted with advanced breast cancer, indicating improved spiritual well-being and psychological adjustment through faith-based interventions (Komariah et al., 2021). Islamic principles provide concrete advantages that enhance emotional resilience and mental well-being, especially in difficult life circumstances.

This model of Islamic psychotherapy, as proposed by Zulkipli et al., incorporates spiritual aspects into psychological treatments. It is claimed that emotional, spiritual, and mental disorders can be effectively treated by methods rooted in Islamic principles, thus linking psychological theories with the spiritual aspects of healing (Zulkipli et al., 2022). This holistic perspective verifies the effectiveness of Islamic teachings in mental wellness and advocates for a combined approach to therapy that takes into account religious faith systems while fostering psychological well-being.

Theological foundations significantly contribute to character development, corresponding closely with the objectives of positive psychology. Early Muslim scholars’ contributions to mental health practices are highlighted by Rothman et al., with Islamic traditions promoting a holistic approach to personal development, encompassing physical, emotional, and spiritual aspects as stated by Rothman et al., (2024). A broad and integrated approach aligns with the principles of positive psychology, which seeks to develop and nurture character strengths that promote resilience and overall well-being.

The Islamic perspective on family dynamics and interpersonal connections reinforces the relevance of positive psychology. Kusumaningrum highlights the importance of compassion, empathy, and balance in family relationships as key components of an individual’s overall well-being, aligning with the principles of positive psychology concerning social support networks (Kusumaningrum, 2023). Familial relationships and responsibilities provide a significant opportunity to boost psychological resilience, suggesting that Islamic teachings can serve as a key basis for developing comprehensive mental health support systems within families.

The interface between Islam and positive psychology provides valuable theoretical perspectives along with practical applications that can guide interventions within multiple areas, such as education, healthcare, and workplaces. Holistic approaches to psychology must incorporate spiritual dimensions, which are essential for fostering resilience, character strengths, and overall well-being, as reinforced by various theoretical connections highlighting the significance of spiritual values.

### Cultural Compatibility of Positive Psychology Practices to Muslim Societies

Islamic teachings place a strong emphasis on the importance of cultural compatibility when considering concepts like resilience and hope, which are viewed as core values for coping with adversity. According to Islamic teachings, the values of “sabr” (patience) and “shukr” (gratitude) are compatible with the principles of positive psychology, which aims to develop resilience and personal strengths (Captari et al., 2022). Integrating these concepts can enhance support systems within Muslim communities, promoting both individual and collective resilience, especially in difficult situations such as post-disaster rehabilitation (Fischer et al., 2010).

Hope is considered vital for well-being in both positive psychology and Islamic philosophy, but Islamic thought places the framework of hope within a spiritual context that is grounded in trust in and reliance on Allah. A theological basis requires a unique conceptual framework for resilience and hope, and interventions should highlight this approach (Captari et al., 2022). This integration can enable a comprehensive wellness model that combines psychological factors with spiritual development, resulting in a more complete understanding of well-being that incorporates both emotional states and spiritual satisfaction.

A challenge in positive psychology may arise from its emphasis on personal growth and independence within culturally individualistic contexts. In contrast, Islamic teachings frequently stress the importance of community, a collective approach, and surrender to divine guidance as essential components of human existence (Achour et al., 2016). Adapting Western psychological practices to Islamic contexts can create tension unless the interventions are tailored to reflect the values of interdependence and social responsibility that are commonly found in many Muslim societies. The difficulty arises from reconciling individual development with communal welfare without compromising the fundamental principles of either entity (Agrawal et al., 2023).

Positive psychology concepts that foster empathy, compassion, and interpersonal connections align with Islamic principles, which encourage kindness and unity among individuals (Surbakti et al., 2024). Incorporating compassionate care and community involvement in interventions can effectively promote mental well-being in Islamic settings (Aldbyani, 2025). Social relationships and community support, a vital component in Islamic traditions, can thus be highlighted in therapeutic approaches, enhancing personal experiences and promoting psychological well-being.

In addition, cultivating character strengths as described in positive psychology aligns with the Islamic tradition of developing moral virtues. The fundamental virtues of honesty, gratitude, and humility are widely acknowledged, which makes their incorporation into positive psychology programs a feasible approach (Qiu et al., 2021). Trying to implement these values within community environments can improve cooperative actions and have a positive impact on the social structure within Muslim communities.

There is a key area where further progress is needed: incorporating spiritual or religious aspects of well-being into psychological therapies. Involving Islamic principles and values in positive psychology approaches is intended to increase adherence and relevance among Muslim communities (Captari et al., 2022). Mental health professionals are encouraged to interact with patients not only as unique individuals, but also as integral parts of their respective faith groups and cultural societies, examining the role spirituality plays in overall well-being.

Given the social and cultural backdrop of Muslim communities, there is a requirement for culturally tailored assessments and instruments that gauge well-being in line with Islamic principles. Current assessment models in positive psychology may neglect crucial cultural subtleties that influence self-perception, resilience, and emotional expression within these communities (Sufya & Abas, 2025). Creating a culturally sensitive framework for evaluating positive psychology concepts is essential to guarantee that interventions align with people’s experiences and goals.

### The Rationale of the Study

This systematic review is being conducted for a variety of reasons, encompassing significant implications across both psychological research and clinical practice, especially in culturally sensitive mental health settings. The underlying premise of this rationale is firmly based on the increasing acknowledgment of the significance of cultural background within the realm of psychological interventions. Previous research has explored various treatment methods, often viewed through a primarily Western perspective, which may not sufficiently consider the distinct cultural and spiritual aspects of Muslim clients (Kiani & Ehsan, 2023). This manuscript emphasizes the importance of therapeutic practices that are both effective and culturally relevant, as shown by studies that synthesize and evaluate positive psychology interventions influenced by Islamic principles (Hendriks et al., 2018). Mental health professionals must prioritize this aspect, as they aim to offer care that considers and values their clients’ belief systems and cultural backgrounds.

The systematic review also underscores the potential advantages of incorporating positive psychological interventions within Islamic contexts. Studies indicate that elements of positive psychology, including resilience, gratitude, and optimism, play a key role in enhancing mental health results across diverse groups (Castro et al., 2020). These positive concepts are especially prominent in Islamic teachings, which emphasize personal development, community assistance, and spiritual health (Casellas‐Grau et al., 2013). By linking positive psychology’s emphasis on strengths and positive qualities with Islamic principles, practitioners can develop interventions that not only ease psychological distress but also foster holistic flourishing among individuals.

The manuscript fills a notable void in existing research by combining the principles of positive psychology with Islamic teachings. A plethora of studies has shown the effectiveness of positive psychology interventions in various Western cultures, yet there is a scarcity of research examining how to effectively design and implement these interventions in Muslim communities (Amonoo et al., 2019). This review methodically examines relevant clinical studies to assess their validity and usability, contributing to the development of a framework for subsequent research that can build upon these preliminary results.

The review also helps inform the creation of evidence-based treatments that are specifically designed for Muslim communities, acknowledging the possibility that traditional therapy methods might not be effective for people who place a strong emphasis on their cultural and spiritual identity in their healing processes (Bérubé et al., 2021). Interventions such as gratitude exercises, resilience training, and acts of kindness become more relatable and effective when framed within the context of Islamic teachings (Imran et al., 2024). Researchers of the examined studies have demonstrated that these practices not only enhance mental well-being, but also promote social unity and facilitate group healing, especially in community-based contexts.

Finally, the manuscript explores the practicability and receptiveness of positive psychology interventions within Muslim communities. When implementing interventions, practitioners must take into account the clients’ beliefs, practices, and individual requirements. Maintaining cultural sensitivity is crucial for building trust and guaranteeing the involvement of patients in therapeutic activities (Hong et al., 2022). The review accordingly recommends preparing mental health professionals to be culturally aware by broadening their knowledge of Islamic principles and practices, which can improve the therapeutic relationship (Amlashi et al., 2024).

## Method

### Search Strategy

A thorough examination of existing literature was undertaken, utilizing electronic databases such as Google Scholar, Web of Science, Scopus, PubMed, and ScienceDirect. The key search terms encompassed “positive psychology,” “intervention,” “Muslim,” “Islam,” “well-being,” and “mental health,” among others related to these concepts. Articles published between 2000 and 2024 were examined, with no geographical limitations. The selection process adhered to the PRISMA (Preferred Reporting Items for Systematic Reviews and Meta-Analyses) guidelines (Page et al., 2021).

### Inclusion and Exclusion Criteria

This comprehensive review comprised studies examining positive psychological interventions in Muslim communities. The specific criteria for selecting the studies included:

- Research examining the implementation of positive psychology interventions among Muslim populations;
- Research examining the intersection of Islamic notions of wellness and evidence-based positive psychology techniques;
- Randomized and non-randomized controlled trials, quasi-experimental studies, and observational studies that have been the focus of empirical research; and
- Research that considered factors like life satisfaction, happiness, resilience, or self-esteem in its psychological outcome assessments.
- Excluded from the study was research that:
- Failed to concentrate on Muslim populations;
- Was not based on empirical evidence, such as theoretical or opinion-based pieces;
- Reported no psychological outcomes associated with positive psychology; and
- Included non-peer-reviewed articles in the analysis.

Finally, we incorporated studies from peer-reviewed publications that aligned with our specified requirements. Studies with missing information, researcher bias, or methodological flaws were filtered out, as were studies with incomplete data, conflicts of interest, or limitations in study design.

### Quality and Risk-of-Bias Assessment

The author evaluated the quality of each study using the Critical Appraisal Skills Program (CASP) checklist tailored for systematic reviews as outlined in CASP (2018). The Measurement Tool to Assess Systematic Reviews (AMSTAR) 2 was evaluated by two separate reviewers, as described by Shea et al. (2017). Each item was carefully evaluated during the scoring process, with statements made that were directly relevant to the items in question. The assessment tools’ findings indicated that this research was a moderate-quality review. The quality assessment and appraisal of the current research were detailed in a supplementary file.

## Results

### Search Results

**Fig. 1:**
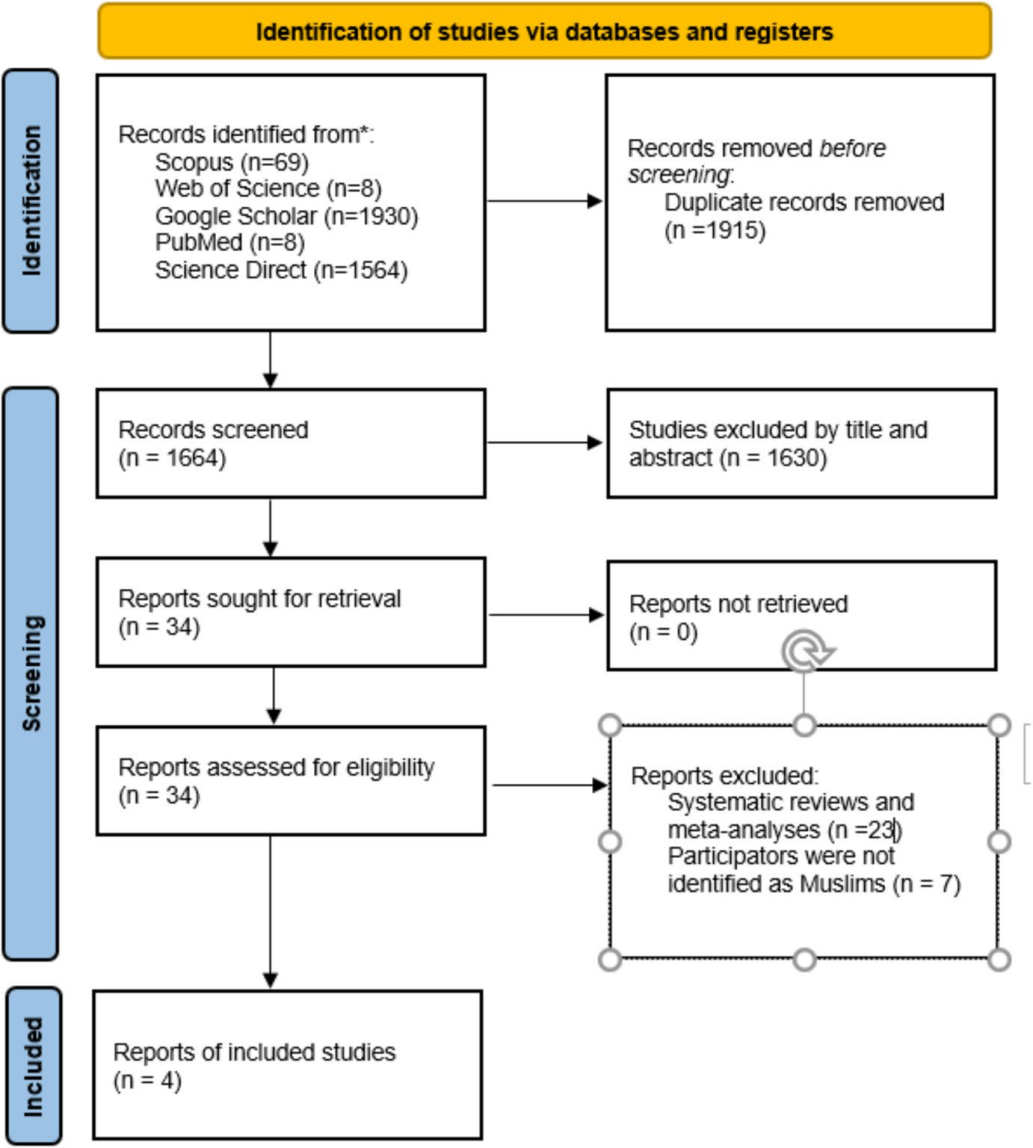
PRISMA flow diagram of studies selected for systematic review

An initial search across six databases resulted in the identification of 3579 articles. After eliminating duplicate entries, a total of 1664 unique records remained. Following the initial review, 1,630 articles were removed due to their titles and abstracts. The eligibility of the remaining 34 articles was assessed based on the specified inclusion and exclusion criteria through a review of their full texts. Subsequently, 30 articles were excluded due to their focus on systematic reviews and meta-analyses (*n* = 23), and because the participants were not identified as Muslim (*n* = 7). As a result, four studies were chosen for in-depth examination. The entire process was illustrated in a PRISMA flow diagram (Fig. 1).

### Study Characteristics

A comprehensive review of existing research found four studies that fit the specified criteria, offering valuable information on the implementation and success of positive psychology interventions within Muslim communities. A table outlining the main features of these studies is provided below to give a comprehensive overview of the research. It contains information on the author and publication date, article location, sample details, groups, intervention type, metrics used, intervention period, and results (Table 1).

**Table 1:** Characteristics of the included studies

Author and date	Geographic location	Sample description	Groups	The type of ıntervention	Measures	Intervention duration	Outcome
Hapsari et al. (2021)	Indonesia	University students (*n* = 10)	Single-group pretest-post-test	Online positive psychology training based on Islamic values (e.g., gratitude, patience, and reliance on God)	Resilience scale	Six sessions	Significant improvement in resilience among the experimental group compared to the control group
Yuliatun and Karyani (2022)	Indonesia	Nurses (*n* = 40. Experimental group = 20, control group = 20)	Experimental and control groups	Islamic positive psychology training (e.g., gratitude, patience, and reliance on God)	Psychological well-being scale	Six sessions	Improved psychological well-being
Basman et al. (2024)	Philippines	Muslim Filipino humanitarian workers exposed to trauma (n = 17)	Single-group pretest–posttest	Islamic psychospiritual module (e.g., prayer, remembrance of God, and forgiveness)	Post-traumatic Growth Inventory (PTGI)	Five modules	Moderate-to-large increases in post-traumatic growth, particularly in personal strength and appreciation for life
Al-Seheel and Noor (2016)	Malaysia	Muslim university students (N = 60); 19 participants in the secular-based exercise group, 20 participants in the Islamic-based exercise group, and 21 participants in the life-detail control group)	Experimental and control groups	Islamic-based gratitude strategy (writing letters expressing gratitude with Quranic references)	The Scale of Positive and Negative Experience (SPANE) and the Satisfaction with Life Scale (SWLS)	3 weeks	Significant increase in happiness levels among the experimental group compared to the control group

The articles were published between the years 2016 and 2024. These studies encompassed a wide range of populations, such as nurses and university students in Indonesia, humanitarian workers in the Philippines, and students in Malaysia. The sample sizes of the studies ranged from 10 to 60. The experimental and control groups, as well as the single-group pretest-post-test design, were employed for the selection of groups. In the intervention type, studies employed a range of Islamic-influenced approaches, including online positive psychology training grounded in Islamic values and three specific components: Islamic positive psychology training, an Islamic psychospiritual module, and an Islamic-based gratitude strategy. A summary of each study’s findings is provided below.

Hapsari et al. (2021) implemented a study to boost resilience in university students via an online training program rooted in Islamic principles. The training was broken down into six sessions, which were conducted online via Zoom, incorporating positive psychology interventions that took into account the cultural and religious backgrounds of the participants. The findings reveal a considerable increase in students’ ability to bounce back from adversity, demonstrating the efficacy of culturally sensitive mental health treatments within school environments. The research conducted by Yuliatun and Karyani in (2022) centered on enhancing the psychological well-being of nurses through a structured training program grounded in Islamic positive psychology principles. The sample consisted of nurses who took part in a 4-week intervention program that employed different psychological well-being assessments. Research revealed that the training had a substantial positive impact on the mental health of the participants, underscoring the significance of including spiritual and cultural factors in mental health strategies for healthcare professionals (Yuliatun & Karyani, 2022).

Researchers led by Basman et al. in (2024) examined the effects of an Islamic psychospiritual program on post-traumatic growth among Muslim aid workers in the Philippines. This pilot study involved an 8-week intervention that aimed to promote resilience and personal growth in individuals who had experienced traumatic events. The findings revealed a positive outcome for post-traumatic growth, implying that incorporating Islamic values into counseling services can have a positive impact on individuals working in high-pressure situations (Basman, 2024). An investigation by Al-Seheel and Noor in (2016) examined the impact of an Islamic-based gratitude approach on the happiness levels of Muslim students residing in Malaysia. The intervention lasted 4 weeks and centered on incorporating gratitude practices consistent with Islamic principles. Research revealed a substantial rise in the happiness levels of the participants, highlighting the efficacy of gratitude as a psychological intervention within the framework of Islamic principles (Al-Seheel & Noor, 2016).

## Discussion

This review emphasizes the potential of positive psychology interventions (PPI) to improve overall well-being within Muslim communities. The four selected studies highlighted the significance of synchronizing PPIs with Islamic values and customs. Studies by Yuliatun and Karyani (2022) and Al-Seheel and Noor (2016) found that incorporating Quranic teachings and Hadiths into interventions increased participant engagement and perceived relevance. Basman et al. discovered in 2024 that spiritual practices like “dhikr” promoted more profound emotional recovery and resilience in people who had experienced trauma. Participants across all studies reported improvements in their psychological well-being, including heightened happiness, decreased stress levels, and increased post-traumatic growth. These findings are consistent with existing evidence demonstrating the universal relevance of positive psychology principles and highlighting the advantage of tailoring interventions to specific cultural contexts.

The four studies examined in the review focus on positive psychology through interventions that utilize Islamic values and principles to improve corresponding psychological outcomes. Hapsari and her co-authors conducted an investigation into online training designed to enhance university students’ resilience, focusing on both positive psychology and Islamic teachings through a six-session structured training program in 2021 (Hapsari et al., 2021). Yuliatun and Karyani investigated the effect of Islamic positive psychology training on the mental health of nurses, showing the relevance of these principles in professional healthcare environments, which is crucial for a frequently overlooked group (Yuliatun & Karyani, 2022).

These studies are supported by their empirical bases. Basman et al. studied the effects of an Islamic psychospiritual module on post-traumatic growth in Muslim humanitarian workers, offering a deeper understanding of psychological recovery after trauma—a subject relevant to both positive psychology and community resilience (Basman, 2024). This enhances the systematic review by focusing on a particularly susceptible subgroup within Muslim communities. Al-Seheel and Noor also examined the impact of an Islamic-based strategy focused on gratitude, discovering a connection between gratitude practices and increased happiness levels among Muslim students, which highlights the significance of incorporating gratitude into daily routines to promote a more positive mental outlook (Al-Seheel & Noor, 2016).

Interventions tailored to Islamic values and customs have been found to be particularly effective. When examining the intersection of positive psychology and psychotherapy in the Muslim community, it is crucial to take into account the specific cultural, religious, and psychological factors that influence mental health interventions (Mudryk & Johnson, 2022; Yaghoobi & Moghadam, 2019). Research into the incorporation of Islamic principles into therapeutic approaches has become increasingly significant, especially given the rising Muslim populations in different parts of the world, including Western regions (Hendriks et al., 2018; Samuels et al., 2021). The current research is synthesized to emphasize the importance of culturally adapted psychotherapy models, the impact of positive psychology interventions, and the difficulties experienced by Muslim individuals in accessing mental health support (Isgandarova, 2014).

Rothman and Coyle highlighted the significance of incorporating Islamic perspectives on psychology into clinical therapy; therapists who comprehend and incorporate Islamic values are better able to meet the needs of their clients (Rothman & Coyle, 2023). Keshavarzi and Haque developed a psychotherapy model to promote Muslim mental health within an Islamic framework; this model suggests therapeutic approaches that respect and consider the spiritual and cultural aspects of clients’ lives (Keshavarzi & Haque, 2013). Mir et al. (2019) emphasize the creation of culturally tailored therapies for Muslim clients suffering from depression, which incorporate positive religious coping mechanisms as a resource for improving well-being. These adaptations do not only enhance treatment results, but also promote a sense of cultural awareness among professionals.

Research has found that positive psychology interventions (PPIs) hold promise for improving well-being within Muslim populations. A study led by Basurrah et al. found that, though the impact of PPIs has been extensively studied in Western countries, they are also effective in Arab nations (Basurrah et al., 2022). According to Alrashdi’s research, cultural adaptations of third-wave psychotherapies, such as positive psychology interventions, are becoming more widely acknowledged as crucial for delivering effective mental health care in countries that belong to the Gulf Cooperation Council (Alrashdi, 2024). By adopting these adaptations, interventions are made more relevant and effective by aligning with the cultural values and religious beliefs of Muslim clients.

### Limitations

The studies yielded important findings; however, it is essential to consider their methodological limitations. The limited generalizability of the results is often attributed to small sample sizes, dependence on self-reported data, and insufficient long-term follow-up periods. Most research focused on particular areas or groups, resulting in a lack of knowledge about how these interventions could be applied to other Muslim communities, including refugees or minority groups residing in Western nations. Mental health issues are often met with mistrust and stigma, as noted by Amri and Bemak in their examination of how these factors affect Muslim immigrants in the US in seeking psychological help (Amri & Bemak, 2013). Furthermore, Rogers-Sirin et al. observe that adopting Western-based psychological practices without considering their cultural implications can result in ineffective treatment options for Muslim communities (Rogers-Sirin et al., 2017). Emphasizing cultural awareness and understanding, mental health professionals need regular education and training to ensure they are equipped to meet the diverse needs of their clients.

When evaluating the generalizability of findings from these studies, it is essential to take into account the variety of populations involved. Hapsari et al.’s study, published in 2021, centers around university students, yielding valuable insights into young adults, yet its results may be restricted when applied to other age groups or cultural settings without additional research (Hapsari et al., 2021). Research conducted by Yuliatun and Karyani, specifically focusing on healthcare professionals, underscores the significance of positive psychology interventions in high-stress professions, though it may not fully encapsulate the experiences of those outside this particular field (Yuliatun & Karyani, 2022).

Basman et al. explore humanitarian workers from a community-focused viewpoint, helping determine how trauma-informed methods can be adapted to meet the needs of various occupational roles, thereby increasing their applicability to others in comparable high-stress work environments (Basman, 2024). Their study by Al-Seheel and Noor yields significant findings relevant to a younger age group, which may be transferable to other educational environments where Islamic principles are a key aspect of student growth (Al-Seheel & Noor, 2016).

These studies, despite their potential limitations in terms of demographic detail, collectively contribute significant insights that highlight the relevance of positive psychology strategies adapted for Islamic settings and traditions. A diverse group of professionals with different ages and experiences can provide a deeper insight into how Muslim communities can utilize positive psychology to tackle their specific difficulties and boost mental health.

### Implications and Recommendations for Research and Practice

The manuscript offers important insights into the use of positive psychology in Islamic settings, highlighting several practical implications and suggestions for psychotherapy practice. This systematic review provides insight into the benefits of combining Islamic principles with positive psychology frameworks for developing mental health interventions targeted at Muslim communities. Existing research provides a foundation for developing culturally sensitive treatments that align with patients’ spiritual principles and priorities, thereby fostering overall mental health and wellness.

The systematic review provides strong evidence supporting the effectiveness of positive psychology interventions tailored to specific cultural and religious backgrounds. Research findings have shown that Islamic-based approaches, such as cultivating gratitude and resilience-building exercises, can notably improve mental health outcomes across diverse groups, including students and healthcare workers (Aprilianti, 2024). This development is particularly significant for psychotherapists working primarily in Muslim-dominated areas, as it enables a therapeutic approach that takes into account and respects the clients’ cultural and spiritual beliefs, thereby enhancing the chances of successful therapeutic outcomes.

In clinical settings, healthcare professionals should contemplate implementing a strengths-based approach that highlights resilience, character strengths, and virtues, as outlined by research studies within Islamic psychology. Therapists should incorporate values such as gratitude and compassion into their therapeutic interventions in a structured manner, enabling them to align with the Islamic values that are highly valued by their clients. A study by Rothman et al. in 2024 indicates that grasping the transformative elements of Islamic teachings can significantly improve the character development process in psychotherapy (Rothman et al., 2024). Psychotherapists should receive training to integrate Islamic principles into their work, creating a setting where patients feel comprehended, valued, and supported in their spiritual paths.

The results of the systematic review indicate that interventions should be assessed not just according to traditional outcomes like symptom relief, but also in relation to spiritual growth, character formation, and adherence to Islamic principles. The data emphasize the significance of enhancing patients’ spiritual well-being concurrently with their mental health (Komariah et al., 2021). Providing interventions like group therapy that incorporates Islamic narratives may improve psychological interventions by enabling clients to better cope with difficulties through more effective coping strategies.

Positive psychology therapists are advised to work in partnership with community organizations and religious leaders when creating interventions and putting them into practice. Collaborative efforts can strengthen the interpersonal aspects of therapy and guarantee that interventions are tailored to the local community, culturally aware, and adaptable, thereby establishing a more extensive support network for individuals receiving treatment.

Mental health training programs should be designed to include Islamic psychology principles and techniques, thereby increasing awareness and comprehension of the importance of incorporating spirituality into therapeutic care. Providing this education can enhance therapeutic relationships, cultural understanding, and ultimately, more effective patient results within Muslim communities (Keshavarzi & Haque, 2013). Carrying out thorough randomized controlled trials to assess the effectiveness of particular PPIs in various Muslim communities is also a significant task. Furthermore, continuous training for mental health professionals on cultural awareness and sensitivity is crucial for enhancing service provision (Theodosopoulos et al., 2024).

Conducting further research into the subtleties of how Islamic values shape psychological characteristics and mental health results is also essential. Research indicates a potential for qualitative investigations into personal experiences with combined psychological methods among those receiving treatment. Adding this information would enhance the knowledge base, enabling ongoing revisions to psychotherapy practices in order to more effectively meet the requirements of community mental health.

It is essential for mental health professionals to identify the specific stressors experienced by Muslim community members, as these may impact their psychological well-being. According to a systematic review, humanitarian workers with a Muslim background may encounter specific types of traumatic experiences that can impact their mental health (Aprilianti, 2024). Interventions tailored to these contextual realities should be implemented, incorporating practices that promote both resilience enhancement and fortification via cultural storytelling and community support systems.

Research in the future should focus on creating and assessing culturally adapted positive psychology interventions for Muslim populations (Mohr et al., 2020). Exploring the effectiveness of targeted interventions that incorporate Islamic principles is essential, particularly for addressing the distinct obstacles faced by Muslims in accessing mental health services (Abu-Ras et al., 2018; Zotova, 2018). Incorporating elements like prayer, scripture reflection, and communal activities rooted in faith can be integrated into various therapeutic approaches. Interventions can be customized to address the specific difficulties encountered by distinct subgroups, like women, young people, or individuals who have survived traumatic experiences. Ultimately, field specialists could investigate how technology can be used to distribute PPIs to a broader range of people within the context of Islamic principles and teachings.

In summary, this systematic review makes significant contributions to the field of psychotherapy by providing a framework that integrates Islamic principles with positive psychological interventions. Mental health professionals working with Muslim communities should prioritize culturally sensitive practices, collaborative partnerships with community members, and ongoing education on Islamic psychology, thereby meeting psychological needs while also fostering spiritual well-being. Recommendations like these create an opportunity for a more comprehensive and encompassing approach to mental health, promoting strength and well-being within Muslim communities.

## Conclusion

In conclusion, integrating positive psychology into psychotherapy for Muslim communities offers a valuable opportunity to enhance mental health outcomes. By considering the cultural and religious factors that influence the experiences of Muslim clients, mental health professionals can develop tailored interventions that promote resilience and overall well-being. Collaboration among mental health practitioners, researchers, and community leaders is essential to address the mental health needs of Muslims and to cultivate a more inclusive and supportive mental health environment.

## Data Availability

Data sharing is not applicable to this article as no new data were created or analyzed in this study.

## References

[CR1] Abdulkerim, N., & Li, C. (2022). How applicable are mindfulness-based interventions to Muslim clients in the U.S.? *Professional Psychology Research and Practice,**53*(3), 253–265. 10.1037/pro0000454

[CR2] Abu-Ras, W., Suárez, Z. E., & Abu-Bader, S. H. (2018). Muslim Americans’ safety and well-being in the wake of Trump: A public health and social justice crisis. *American Journal of Orthopsychiatry,**88*(5), 503–515. 10.1037/ort0000321 29629779 10.1037/ort0000321

[CR3] Achour, M., Bensaid, B., & Nor, M. R. B. M. (2016). An Islamic perspective on coping with life stressors. *Applied Research in Quality of Life,**11*(3), 663–685. 10.1007/s11482-015-9389-8

[CR4] Agrawal, J., Singh, K., & Saxena, G. (2023). Religion, spirituality, and happiness through an Indian lens. In K. Singh & G. Saxena (Eds.), *Religious and spiritual practices in India: A positive psychological perspective. *Springer Nature.

[CR6] Aldbyani, A. (2025). Exploring Islamic mindfulness: Cultural practices and their impact on public health outcomes. *Mindfulness,**16*(3), 695–701. 10.1007/s12671-024-02485-5

[CR7] Alhomaizi, D., Alsaidi, S., Moalie, A., Muradwij, N., Borba, C. P., & Lincoln, A. K. (2018). An exploration of the help-seeking behaviors of Arab-Muslims in the US: A socio-ecological approach. *Journal of Muslim Mental Health*. 10.3998/jmmh.10381607.0012.102

[CR8] Ali, S., Mahmood, A., McBryde-Redzovic, A., Humam, F., & Awaad, R. (2022). Role of mosque communities in supporting Muslims with mental illness: Results of CBPR-oriented focus groups in the Bay Area California. *Psychiatric Quarterly,**93*(4), 985–1001. 10.1007/s11126-022-10002-x 36322232 10.1007/s11126-022-10002-x PMC9628571

[CR9] Ali, S., Mahoui, I., Hassoun, R., Mojaddidi, H., & Awaad, R. (2023). The Bay Area Muslim Mental Health Community Advisory Board: Evaluation of a community based participatory approach. *Epidemiology and Psychiatric Sciences*. 10.1017/s2045796022000786 10.1017/S2045796022000786 PMC9971854 36718769

[CR10] Alrashdi, D. H. (2024). Cultural adaptations of third-wave psychotherapies in Gulf Cooperation Council Countries: A systematic review. *Transcultural Psychiatry,**61*(2), 209–228. 10.1177/13634615241227691 10.1177/13634615241227691 PMC10943625 38332485

[CR11] Al-Seheel, A. Y., & Noor, N. M. (2016). Effects of an Islamic-based gratitude strategy on Muslim students’ level of happiness. *Mental Health, Religion & Culture,**19*(7), 686–703. 10.1080/13674676.2016.1229287

[CR12] Altalib, H., Elzamzamy, K., Fattah, M., Ali, S., & Awaad, R. (2019). Mapping global Muslim mental health research: Analysis of trends in the English literature from 2000 to 2015. *Cambridge Prisms Global Mental Health*. 10.1017/gmh.2019.3 31157114 10.1017/gmh.2019.3 PMC6533849

[CR14] Amlashi, R. S., Majzoobi, M., & Forstmeier, S. (2024). The relationship between acculturative stress and psychological outcomes in international students: A systematic review and meta-analysis. *Frontiers in Psychology*. 10.3389/fpsyg.2024.1403807 10.3389/fpsyg.2024.1403807 PMC11253713 39021659

[CR15] Amonoo, H. L., Barclay, M. E., El-Jawahri, A., Traeger, L., Lee, S. J., & Huffman, J. C. (2019). Positive psychological constructs and health outcomes in hematopoietic stem cell transplantation patients: A systematic review. *Biology of Blood and Marrow Transplantation*. 10.1016/j.bbmt.2018.09.030 10.1016/j.bbmt.2018.09.030 30308327

[CR16] Amri, S., & Bemak, F. (2013). Mental health help-seeking behaviors of Muslim immigrants in the United States: Overcoming social stigma and cultural mistrust. *Journal of Muslim Mental Health*. 10.3998/jmmh.10381607.0007.104

[CR17] Aprilianti, E. (2024). Integrating Islamic psychological principles in enhancing students’ academic resilience. *Nusantara Journal of Behavioral and Social Sciences,**3*(2), 63–72. 10.47679/202246

[CR18] Arfken, C. L., & Ahmed, S. (2016). Ten years of substance use research in Muslim populations: Where do we go from here? *Journal of Muslim Mental Health*. 10.3998/jmmh.10381607.0010.103

[CR20] Bagasra, A., & Mackinem, M. (2014). An exploratory study of American Muslim conceptions of mental illness. *Journal of Muslim Mental Health*. 10.3998/jmmh.10381607.0008.104

[CR21] Basman, A. T. S. (2024). The impact of the Islamic psychospiritual module on posttraumatic growth in Muslim Filipino humanitarian workers: A pilot study. International Journal of Academic Research for Humanities. 10.5281/zenodo.14094068

[CR22] Basurrah, A. A., Blasi, Z. D., Lambert, L., Murphy, M., Warren, M. A., Setti, A., Baddar, M. A., & Shrestha, T. (2022). The effects of positive psychology interventions in Arab Countries: A systematic review. *Applied Psychology Health and Well-Being,**15*(2), 803–821. 10.1111/aphw.12391 10.1111/aphw.12391 35856920

[CR23] Basurrah, A. A., Lambert, L., Setti, A., Murphy, M., Warren, M. A., Shrestha, T., & Blasi, Z. D. (2021). Effects of positive psychology interventions in Arab countries: A protocol for a systematic review. *British Medical Journal Open,**11*(7), e052477. 10.1136/bmjopen-2021-052477 10.1136/bmjopen-2021-052477 PMC8323366 34326058

[CR24] Bérubé, M., Martorella, G., Côté, C., Gélinas, C., Feeley, N., Choinière, M., Parent, S., & Streiner, D. L. (2021). The effect of psychological interventions on the prevention of chronic pain in adults. *Clinical Journal of Pain,**37*(5), 379–395. 10.1097/ajp.0000000000000922 33577194 10.1097/AJP.0000000000000922

[CR25] Captari, L. E., Sandage, S. J., & Vandiver, R. A. (2022). Spiritually integrated psychotherapies in real-world clinical practice: Synthesizing the literature to identify best practices and future research directions. *Psychotherapy,**59*(3), 307–320. 10.1037/pst0000407 10.1037/pst0000407 34843316

[CR26] Casellas-Grau, A., Font, A., & Vives, J. (2013). Positive psychology interventions in breast cancer. *A Systematic Review. Psycho-Oncology,**23*(1), 9–19. 10.1002/pon.3353 10.1002/pon.3353 23897834

[CR27] Casey, S., Moss, S., & Wicks, J. (2020). Exploring the accessibility of child-centered play therapy for Australian Muslim children. *Journal of Cross-Cultural Psychology,**51*(3–4), 241–259. 10.1177/0022022120913117

[CR28] CASP, U. (2018). CASP systematic review checklist. *CASP UK*. https://casp-uk.net/casp-tools-checklists/systematic-review-checklist/

[CR29] Castro, M. M. L., Ferreira, R. O., Fagundes, N. C. F., Almeida, A. P. C. P. S. C., Maia, L. C., & Lima, R. R. (2020). Association between psychological stress and periodontitis: A systematic review. *European Journal of Dentistry,**14*(01), 171–179. 10.1055/s-0039-1693507 32069501 10.1055/s-0039-1693507 PMC7069755

[CR32] Diaków, D. M., & Goforth, A. N. (2021). Supporting Muslim refugee youth during displacement: Implications for international school psychologists. *School Psychology International,**42*(3), 238–258. 10.1177/0143034320987280

[CR33] DuPont, C. M., Pressman, S. D., Reed, R., Manuck, S. B., Marsland, A. L., & Gianaros, P. J. (2022). Does an online positive psychological intervention improve positive affect in young adults during the COVID-19 pandemic? *Affective Science,**4*(1), 101–117. 10.1007/s42761-022-00148-z 10.1007/s42761-022-00148-z PMC9589760 36311219

[CR34] Feizi, M. (2024). Effect of positive psychology online group therapy on spiritual well-being, positive and negative affect of working women in COVID-19 pandemic. *Iranian Journal of Psychiatry and Behavioral Sciences*. 10.5812/ijpbs-138380

[CR35] Fischer, P., Ai, A. L., Aydin, N., Frey, D., & Haslam, S. A. (2010). The relationship between religious identity and preferred coping strategies: An examination of the relative importance of interpersonal and intrapersonal coping in Muslim and Christian faiths. *Review of General Psychology,**14*(4), 365–381. 10.1037/a0021624

[CR38] Han, H., Lee, S., Ariza-Montes, A., Al-Ansi, A., Tariq, B., Vega-Muñoz, A., & Park, S. (2021). Muslim travelers’ inconvenient tourism experience and self-rated mental health at a Non-Islamic country: Exploring gender and age differences. *International Journal of Environmental Research and Public Health,**18*(2), 758. 10.3390/ijerph18020758 33477400 10.3390/ijerph18020758 PMC7829772

[CR39] Hapsari, D. K., Karyani, U., & Hertinjung, W. (2021). Positive psychology online training based on Islamic value to improve student resilience. *Indigenous: Jurnal Ilmiah Psikologi,**6*(3), 36–48. 10.23917/indigenous.v6i3.14043

[CR40] Haque, A., Khan, F., Keshavarzi, H., & Rothman, A. E. (2016). Integrating Islamic traditions in modern psychology: Research trends in last ten years. *Journal of Muslim Mental Health,**10*(1), 75–100. 10.3998/jmmh.10381607.0010.107

[CR41] Hassan, M. K. (2021). Contemporary psychological disorders and the spiritual therapy from the Qur’an and the Sunnah. *Revelation and Science*. 10.31436/revival.v11i1.271

[CR42] Hendriks, T., Schotanus-Dijkstra, M., Hassankhan, A., Graafsma, T., Bohlmeijer, E. T., & Jong, J. D. (2018). The efficacy of positive psychology interventions from Non-Western countries: A systematic review and meta-analysis. *International Journal of Wellbeing,**8*(1), 71–98. 10.5502/ijw.v8i1.711

[CR43] Ho, H. C., Mui, M., Wan, A., Ng, Y.-l, Stewart, S. M., Yew, C., Lam, T. H., & Chan, S. S. (2016a). Happy family kitchen: A community-based research for enhancing family communication and well-being in Hong Kong. *Journal of Family Psychology,**30*(6), 752–762. 10.1037/fam0000233 27513284 10.1037/fam0000233

[CR44] Ho, H. C., Mui, M., Wan, A., Ng, Y.-l, Stewart, S. M., Yew, C., Lam, T. H., & Chan, S. S. (2016b). Happy family kitchen II: A cluster randomized controlled trial of a community-based positive psychology family intervention for subjective happiness and health-related quality of life in Hong Kong. *Trials*. 10.1186/s13063-016-1508-9 27473842 10.1186/s13063-016-1508-9 PMC4966601

[CR46] Hong, H. C., Min, A., & Kim, Y. M. (2022). A systematic review and pooled prevalence of symptoms among childhood and adolescent and young adult cancer survivors. *Journal of Clinical Nursing,**32*(9–10), 1768–1794. 10.1111/jocn.16201 35014094 10.1111/jocn.16201

[CR47] Imran, A., Tariq, S., Kapczinski, F., & Cardoso, T. D. (2024). Psychological resilience and mood disorders: A systematic review and meta-analysis. *Trends in Psychiatry and Psychotherapy*. 10.47626/2237-6089-2022-0524 36215270 10.47626/2237-6089-2022-0524 PMC11332678

[CR48] Isgandarova, N. (2014). The evolution of Islamic spiritual care and counseling in Ontario in the context of the college of registered psychotherapists and registered mental health therapists of Ontario. *Journal of Psychology & Psychotherapy*. 10.4172/2161-0487.1000143

[CR49] Keshavarzi, H., & Haque, A. (2013). Outlining a psychotherapy model for enhancing Muslim mental health within an Islamic context. *International Journal for the Psychology of Religion,**23*(3), 230–249. 10.1080/10508619.2012.712000

[CR50] Khait, A. A., & Lazenby, M. (2021). Psychosocial-spiritual interventions among Muslims undergoing treatment for cancer: An integrative review. *BMC Palliative Care*. 10.1186/s12904-021-00746-x 33781246 10.1186/s12904-021-00746-x PMC8008674

[CR51] Kiani, F. S., & Ehsan, S. (2023). Association of positive psychological factors with the mental health of older adult retirees: A systematic review. *International Journal of Human Rights in Healthcare,**17*(5), 505–519. 10.1108/ijhrh-12-2022-0133

[CR52] Komariah, M., Qadous, S. G., Firdaus, M. K. Z. H., Agustina, H. R., Mediawati, A. S., Yulianita, H., Praptiwi, A., Setyorini, D., & Permana, R. H. (2021). The psychological experiences of using Islamic philosophy approach among women’s with advanced breast cancer in Indonesia. *Open Access Macedonian Journal of Medical Sciences,**9*(6), 133–137. 10.3889/oamjms.2021.7320

[CR53] Kusumaningrum, F. A. (2023). The meaning of verses on parents-children relationship as basis for sandwich generation concept in Islam. *Millah: Journal of Religious Studies,**22*(2), 553–582. 10.20885/millah.vol22.iss2.art10

[CR54] Lowe, S. R., Tineo, P., Bonumwezi, J., & Bailey, E. (2019). The trauma of discrimination: Posttraumatic stress in Muslim American college students. *Traumatology an International Journal,**25*(2), 115–123. 10.1037/trm0000197

[CR55] Martin, M. B. (2015). Perceived discrimination of Muslims in health care. *Journal of Muslim Mental Health*. 10.3998/jmmh.10381607.0009.203

[CR56] Mir, G., Ghani, R., Meer, S., & Hussain, G. (2019). Delivering a culturally adapted therapy for Muslim clients with depression. *The Cognitive Behaviour Therapist*. 10.1017/s1754470x19000059

[CR57] Mitchell, J., Vella-Brodrick, D., & Klein, B. (2010). Positive psychology and the internet: A mental health opportunity. *E-Journal of Applied Psychology,**6*(2), 30–41. 10.7790/ejap.v6i2.230

[CR58] Mitha, K. (2020). Conceptualising and addressing mental disorders amongst Muslim communities: Approaches from the Islamic golden age. *Transcultural Psychiatry,**57*(6), 763–774. 10.1177/1363461520962603 33059527 10.1177/1363461520962603 PMC7689558

[CR59] Mohr, S., Shaiq, S., & Berte, D. Z. (2020). Directive vs. non-directive clinical approaches: Liberation psychology and Muslim mental health. *Journal of Islamic Faith and Practice,**3*(1), 31–58. 10.18060/24667

[CR60] Montiel, C., Radziszewski, S., Prilleltensky, I., & Houle, J. (2021). Fostering positive communities: A scoping review of community-level positive psychology interventions. *Frontiers in Psychology,**12*, 1–12. 10.3389/fpsyg.2021.720793 10.3389/fpsyg.2021.720793 PMC8488140 34616336

[CR61] Mudryk, E. P., & Johnson, L. R. (2022). The impact of religiousness and beliefs about mental illness on help-seeking behaviors of Muslim Americans. *Journal of Clinical Psychology,**79*(4), 1208–1222. 10.1002/jclp.23466 36465019 10.1002/jclp.23466

[CR62] Muller-Dugic, J. (2024). A positive psychology intervention to enhance mental well-being of Syrian refugees in the Netherlands. *International Journal of Migration Health and Social Care,**20*(1), 104–124. 10.1108/ijmhsc-07-2022-0074

[CR63] Mullick, M. S. I., Khalifa, N., Nahar, J. S., & Walker, D. (2013). Beliefs about Jinn, black magic and evil eye in Bangladesh: The effects of gender and level of education. *Mental Health Religion & Culture,**16*(7), 719–729. 10.1080/13674676.2012.717918

[CR65] Nadir, P. A. (2024). Muslim American families: A social worker’s reflections. *Journal of Islamic Faith and Practice,**5*(1), 97–113. 10.18060/28096

[CR66] Omu, O., Al-Obaidi, S., & Reynolds, F. (2012). Religious faith and psychosocial adaptation among stroke patients in Kuwait: A mixed method study. *Journal of Religion and Health,**53*(2), 538–551. 10.1007/s10943-012-9662-1 10.1007/s10943-012-9662-1 23143113

[CR95] Page, M. J., McKenzie, J. E., Bossuyt, P. M., Boutron, I., Hoffmann, T. C., Mulrow, C. D., Shamseer, L., Tetzlaff, J. M., Akl, E. A., Brennan, S. E., Chou, R., Glanville, J., Grimshaw, J. M., Hróbjartsson, A., Lalu, M. M., Li, T., Loder, E. W., Mayo-Wilson, E., McDonald, S., McGuinness, L. A., Stewart, L.A., Thomas, J., Tricco, A.C., Welch, V.A., Whiting, P., & Moher, D. (2021). The PRISMA 2020 statement: an updated guideline for reporting systematic reviews. *BMJ,**372*. 10.1136/bmj.n71 10.1136/bmj.n71 PMC8005924 33782057

[CR67] Qiu, Y., Huang, Y., Wang, Y., Ren, L., Jiang, H., Zhang, L., & Dong, C. (2021). The role of socioeconomic status, family resilience, and social support in predicting psychological resilience among Chinese maintenance hemodialysis patients. *Frontiers in Psychiatry*. 10.3389/fpsyt.2021.723344 34658959 10.3389/fpsyt.2021.723344 PMC8514615

[CR70] Rogers-Sirin, L., Yanar, C., Yüksekbaş, D., Senturk, M. I., & Şirin, S. R. (2017). Religiosity, cultural values, and attitudes toward seeking psychological services in Turkey. *Journal of Cross-Cultural Psychology,**48*(10), 1587–1604. 10.1177/0022022117732532

[CR71] Rothman, A., & Coyle, A. (2023). The clinical scope of Islamic psychotherapy: A grounded theory study. *Spirituality in Clinical Practice,**10*(1), 4–19. 10.1037/scp0000282

[CR72] Rothman, A., Yücesoy, Z. B., & Yalçın, E. (2024). Early Muslim scholars’ conceptions of character development and contemporary applications in mental health and well-being. *Journal of Muslim Mental Health*. 10.3998/jmmh.6027

[CR73] Sabry, W., & Vohra, A. (2013). Role of Islam in the management of psychiatric disorders. *Indian Journal of Psychiatry,**55*(6), 205. 10.4103/0019-5545.105534 10.4103/0019-5545.105534 PMC3705684 23858256

[CR74] Saleem, M. Q., Bakar, A., Durrani, A. K., & Manzoor, Z. (2021). Impact of perceived severity of COVID-19 (SARS-COV-2) on mental health of university students of Pakistan: The mediating role of Muslim religiosity. *Frontiers in Psychiatry*. 10.3389/fpsyt.2021.560059 34408670 10.3389/fpsyt.2021.560059 PMC8365036

[CR75] Samuels, E. A., Orr, L., White, E. B., Saadi, A., Padela, A. I., Westerhaus, M., Bhatt, A. D., Agrawal, P., Wang, D., & Gonsalves, G. (2021). Health care utilization before and after the “Muslim ban” executive order among people born in Muslim-Majority countries and living in the US. *Jama Network Open,**4*(7), e2118216. 10.1001/jamanetworkopen.2021.18216 34328502 10.1001/jamanetworkopen.2021.18216 PMC8325073

[CR76] Saritoprak, S. N., & Abu-Raiya, H. (2023). Living the good life: An Islamic perspective on positive psychology. In E. B. Davis, E. L. Worthington Jr., & S. A. Schnitker (Eds.), *Handbook of positive psychology, religion, and spirituality* (pp. 179–193). Springer Nature.

[CR77] Shea, B. J., Reeves, B. C., Wells, G., Thuku, M., Hamel, C., Moran, J., Moher, D., Tugwell, P., Welch, V., Kristjansson, E., & Henry, D. A. (2017). AMSTAR 2: A critical appraisal tool for systematic reviews that include randomised or non-randomised studies of healthcare interventions, or both. *BMJ*. 10.1136/bmj.j4008 10.1136/bmj.j4008 PMC5833365 28935701

[CR78] Sufya, D. H., & Abas, N. A. H. (2025). Exploring life satisfaction as a bridge between taqwa and psychological well-being in Muslim adolescents. *International Journal of Islamic Educational Psychology,**5*(2), 337–355. 10.18196/ijiep.v5i2.24976

[CR79] Surbakti, S. S. B., Harahap, R., & Hasanah, U. (2024). Future perspectives on the Islamic personality model: Integrating spiritual, moral, intellectual, social, personal, and behavioral dimensions for holistic development. *Journal on Islamic Studies,**1*(1), 17–35. 10.35335/7adqms82

[CR80] Tan, Q. (2023). Analysis of the effect of positive mental group counseling for college students based on PERMA model. *Academic Journal of Humanities & Social Sciences*. 10.25236/ajhss.2023.060103

[CR81] Tangsangwornthamma, C., Ahmad, N., & Rattanamongkolgul, S. (2018). A qualitative study on belief, perception, and health effects on standing zikr among Thai Muslims in Nakhon Nayok province. *Journal of Psychology Research*. 10.17265/2159-5542/2018.11.002

[CR82] Tanhan, A. (2019). Acceptance and commitment therapy with ecological systems theory: Addressing Muslim mental health issues and wellbeing. *Journal of Positive School Psychology,**3*(2), 197–219. 10.47602/jpsp.v3i2.172

[CR83] Tanhan, A., & Francisco, V. T. (2019). Muslims and mental health concerns: A social ecological model perspective. *Journal of Community Psychology,**47*(4), 964–978. 10.1002/jcop.22166 30730559 10.1002/jcop.22166

[CR85] Theodosopoulos, L., Fradelos, E. C., Panagiotou, A., Dreliozi, A., & Tzavella, F. (2024). Delivering culturally competent care to migrants by healthcare personnel: A crucial aspect of delivering culturally sensitive care. *Social Sciences,**13*(10), 530. 10.3390/socsci13100530

[CR86] Thomas, J., & Barbato, M. (2020). Positive religious coping and mental health among Christians and Muslims in response to the COVID-19 pandemic. *Religions,**11*(10), 498. 10.3390/rel11100498

[CR87] Uddin, M. F., Williams, A., & Alcock, K. (2022). Visibility as Muslim, perceived discrimination and psychological distress among Muslim students in the UK. *Journal of Muslim Mental Health*. 10.3998/jmmh.135

[CR89] Yaghoobi, A., & Moghadam, B. N. (2019). The effect of positive psychology intervention on the psychological well-being of adolescents. *Iranian Journal of Psychiatry and Clinical Psychology,**25*(1), 14–25. 10.32598/ijpcp.25.1.14

[CR91] Yuliatun, I., & Karyani, U. (2022). Improving the psychological well-being of nurses through Islamic positive psychology training. *Psikohumaniora: Jurnal Penelitian Psikologi,**7*(1), 91–102. 10.21580/pjpp.v7i1.10792

[CR92] Yurayat, P., & Seechaliao, T. (2021). Effectiveness of online positive psychology intervention on psychological well-being among undergraduate students. *Journal of Education and Learning,**10*(4), 143. 10.5539/jel.v10n4p143

[CR93] Zotova, N. (2018). Religion and mental health among Central Asian Muslim immigrants in Chicago metropolitan area. *Migration Letters,**15*(3), 361–376. 10.33182/ml.v15i3.358

[CR94] Zulkipli, S. N., Suliaman, I., Abidin, M. S. Z., Anas, N., & Ahmat, A. C. (2022). The development theory of Al-‘Aql, Al-Qalb and al-Nafs in Islamic psychotherapy. *International Journal of Academic Research in Business and Social Sciences,**12*(11), 2432–2449. 10.6007/ijarbss/v12-i11/15013

